# Re-Audit of Documentation Practices in the Blossomwood Electroconvulsive Therapy Suite Using a Quality Improvement Approach

**DOI:** 10.1192/bjo.2026.11406

**Published:** 2026-06-30

**Authors:** Oluwatosin Kadri, Deepa Krishnan, Michael Rajendram

**Affiliations:** Nottinghamshire Healthcare NHS Foundation Trust, Nottingham, United Kingdom

## Abstract

**Aims:**

To assess the completeness and quality of ECT documentation across seven patient records and to implement improvements where compliance fell below 100%.

**Methods:**

A retrospective review of seven ECT cases conducted between October and December 2024 was performed. Each case was evaluated against a 20-item ECT documentation checklist. Compliance rates were calculated, and deficiencies were examined using a QI framework to determine underlying causes.

**Results:**

Full Compliance (100%) was achieved in 15 of 20 criteria. Deficiencies were noted in the documentation of Montgomery–Åsberg depression rating scale (MADRS) score (71.4%), Mini-Addenbrooke's Cognitive Examination (MINI-ACE) assessments (85.7%), and record of previous treatment failure (85.7%). Root causes included limited staff awareness, non-mandatory templates, unclear policies and reliance on paper-based systems.

Two of the five non-compliant criteria related to capacity and consent, in both cases, documentation accurately reflected the clinical situation and were therefore considered fully compliant.

**Conclusion:**

While overall documentation standards were high,notable gaps persisted in clinical assessment domains. A structured QI approach, combining system redesign with enhanced staff support has the potential to address these deficiencies and strengthen the safety, consistency and governance of ECT delivery.

